# The differential role of CR3 (CD11b/CD18) and CR4 (CD11c/CD18) in the adherence, migration and podosome formation of human macrophages and dendritic cells under inflammatory conditions

**DOI:** 10.1371/journal.pone.0232432

**Published:** 2020-05-04

**Authors:** Szilvia Lukácsi, Tamás Gerecsei, Katalin Balázs, Barbara Francz, Bálint Szabó, Anna Erdei, Zsuzsa Bajtay

**Affiliations:** 1 MTA-ELTE Immunology Research Group, Eötvös Loránd University, Budapest, Hungary; 2 Department of Biological Physics, Eötvös Loránd University, Budapest, Hungary; 3 Nanobiosensorics “Lendület” Group, Institute of Technical Physics and Material Sciences, Centre for Energy Research, Hungarian Academy of Sciences, Budapest, Hungary; 4 Department of Immunology, Eötvös Loránd University, Budapest, Hungary; 5 CellSorter Company for Innovations, Budapest, Hungary; Thomas Jefferson University, UNITED STATES

## Abstract

CR3 and CR4, the leukocyte specific β_2_-integrins, involved in cellular adherence, migration and phagocytosis, are often assumed to have similar functions. Previously however, we proved that under physiological conditions CR4 is dominant in the adhesion to fibrinogen of human monocyte-derived macrophages (MDMs) and dendritic cells (MDDCs). Here, using inflammatory conditions, we provide further evidence that the expression and function of CR3 and CR4 are not identical in these cell types. We found that LPS treatment changes their expression differently on MDMs and MDDCs, suggesting a cell type specific regulation. Using mAb24, specific for the high affinity conformation of CD18, we proved that the activation and recycling of β_2_-integrins is significantly enhanced upon LPS treatment. Adherence to fibrinogen was assessed by two fundamentally different approaches: a classical adhesion assay and a computer-controlled micropipette, capable of measuring adhesion strength. While both receptors participated in adhesion, we demonstrated that CR4 exerts a dominant role in the strong attachment of MDDCs. Studying the formation of podosomes we found that MDMs retain podosome formation after LPS activation, whereas MDDCs lose this ability, resulting in a significantly reduced adhesion force and an altered cellular distribution of CR3 and CR4. Our results suggest that inflammatory conditions reshape differentially the expression and role of CR3 and CR4 in macrophages and dendritic cells.

## Introduction

The complement receptors CR3 (CD11b/CD18, also known as Mac-1; α_M_β_2_) and CR4 (CD11c/CD18, also known as p150,95; α_X_β_2_) belong to the family of β_2_-integrins and play an important role in phagocytosis, cellular adherence and migration [1]. Their ligands include iC3b, the activation product of complement component C3, present on opsonized targets, as well as the adhesion ligands fibrinogen and ICAM-1 [2–4]. The ligand binding affinity of integrins is regulated by activation dependent conformational changes. Their extracellular domains undergo remarkable structural rearrangements during the switch from a bent, inactive state into an extended, ligand-binding conformation [5,6].

Based on findings showing that CR3 and CR4 have overlapping ligand binding specificity and share 87% sequence homology in their extracellular domains [7], these two receptors are generally assumed to exert similar functions. However, their intracellular tails, important for signal transduction and connection with the cytoskeleton, markedly differ in length and amino acid sequence—displaying only 56% similarity [8] -, suggesting distinctive functions for these receptors. Our group was the first to comprehensively study the individual role of CR3 and CR4 in various functions of different human phagocytes [9,10]. We proved that there is a “division of labor” between these two receptors under physiological conditions. Namely, we demonstrated that CR3 is in control of the phagocytosis of iC3b opsonized bacteria while CR4 dominates cell adhesion to fibrinogen [11–13].

Fibrinogen, a major ligand of β_2_-integrins, is an acute phase reactant, which is a key regulator of inflammation in disease [14]. It deposits at the sites of injury and contributes to the inflammatory response by participating in the adhesion and migration of leukocytes. By their interaction with fibrinogen [15,16], CR3 and CR4 are known to facilitate cell activation, cytokine and chemokine production [17,18]. Although an elevated expression of CR3 and CR4 has been observed in pathological conditions [19,20], their exact role in human macrophages and dendritic cells has not been studied in detail under inflammatory conditions. The lack of this knowledge prompted us to investigate the adhesive and migratory function of these β_2_-integrins in the inflammatory response induced by LPS.

Myeloid cells achieve movement by forming podosomes, that are adhesive structures having an F-actin core surrounded by adhesion molecules, like integrins [21,22]. Podosomes also sense the rigidity and structure of their environment, and help cell progression through the degradation of matrix components with matrix metalloproteinases and ADAMs (a disintegrin and metalloproteinase) [23,24]. The crucial role of β_2_-integrins in podosome formation is well established [25,26] and our group also showed earlier that both CR3 and CR4 are present in the adhesion ring of podosomes formed by monocyte-derived macrophages (MDMs) and dendritic cells (MDDCs) on a fibrinogen coated surface [12].

Recent studies have shown, that M1 macrophages–i.e. cells activated by LPS and IFNγ - express CCR7 and migrate in the direction of CCL19 and CCL21 chemokine gradient [27], which results in their accumulation at the inflammatory sites [28,29]. Dendritic cells are known to migrate to the lymph nodes after antigen uptake, and during this journey they go through a maturation process [30]. Maturation induces changes in chemokine receptor expression [31], including CCR7, which appears 3 hours after the inflammatory stimulus, and becomes more pronounced after 12–24 hours [32]. The maturation of dendritic cells is also accompanied by dynamic changes in the actin cytoskeleton, that entails decreased phagocytosis and the loss of podosome formation [25,33,34].

Our group set out to thoroughly investigate how CR3 and CR4 participate in leukocyte functions necessary for the resolution of infections. To better understand these processes, our aim was to reveal the role of CR3 and CR4 in adhesion and migration during the inflammatory response and to extend these studies by investigating the force of adhesion and the distribution of CR3 and CR4 in the contact zone of macrophages and dendritic cells after LPS-treatment.

## Materials and methods

### Isolation of human monocytes

Monocytes were isolated from buffy coat obtained from healthy donors and delivered by the Hungarian National Blood Transfusion Service. Informed consent was provided for the use of blood samples. The study was carried out in accordance with the Helsinki Declaration and was approved by the Ethics Committee of the Medical Research Council in Hungary (TUKEB), 52088/2015/EKU. Peripheral blood mononuclear cells (PBMC) were separated by Ficoll-Paque PLUS (GE Healthcare Bio-Science, Uppsala, Sweden) density gradient centrifugation and monocytes were isolated using the Miltenyi CD14 MicroBeads (Miltenyi Biotech, Bergisch Gladbach, Germany). Purity of isolated monocytes was analyzed by flow cytometry using anti-CD14 antibody (ImmunoTools GmbH, Friesoythe, Germany).

### Generation of MDMs and MDDCs

Monocytes were cultivated in RPMI-1640 Medium (Sigma-Aldrich Inc., St Louis, MO, USA) supplemented with gentamicin (Sigma-Aldrich) and 10% FCS (Sigma-Aldrich). To generate MDMs 40 ng/ml rHu GM-CSF (R&D systems, Minneapolis, USA) was added to the isolated monocytes. To generate MDDCs 40 ng/ml rHu GM-CSF (R&D systems) and 15 ng/ml rHu IL-4 (R&D systems) were added to the monocytes. Cytokines were resupplied on day 3 of differentiation. Studies were carried out on day 5.

### Induction of inflammatory condition using LPS treatment

MDMs and MDDCs were treated with 100 ng/ml bacterial lipopolysaccharide (LPS, Sigma-Aldrich) on the 5^th^ day of differentiation to induce cell activation. Cells were cultured in 12-well cell culture plates at 10^6^ cells/ml with LPS or without LPS (untreated control) for different time periods.

### Monitoring the expression of CD11b and CD11c during LPS induced activation

The expression of CD11b and CD11c was measured by flow cytometry at different time points during the LPS induced cell activation. LPS treated and untreated control cells were harvested after 30 minutes, 24 hours or 48 hours, and washed with ice cold PBS (phosphate buffered saline solution) supplemented with 0.4% sodium azide and 1% FCS. Cells were labelled with anti-CD11b-FITC and anti-CD11c-APC (ImmunoTools GmbH) for 20 minutes on ice. The expression of the receptors was compared to the appropriate untreated control sample at each time point. Samples were analyzed on Cytoflex flow cytometer (Beckman Coulter) using CytExpert software for data acquisition and Kaluza Analysis software for data analysis.

### Analysis of β_2_-integrin conformational state

The conformation state of β_2_-integrins was determined using the activation epitope specific mouse monoclonal mAb24 antibody labelled with Alexa488 (BioLegend, San Diego, California, USA). 5x10^5^ cells were resuspended in 400 μl of RPMI-1640 medium and labelled with mAb24-Alexa488 antibody for 20 minutes on ice under sterile conditions. Without washing, the tubes were moved to a 37°C CO_2_ incubator or left on ice (0-minute controls). To induce activation, 100 ng/ml LPS was added to the cells at the beginning of the 37°C incubation. After the incubation time cells were washed with ice cold PBS supplemented with 0.4% sodium azide and 1% FCS and put on ice immediately, to stop the receptor internalization. Samples were analyzed on Cytoflex flow cytometer at 0 minutes and 30 minutes. Confocal microscopy images were prepared at 0, 5 and 30 minutes by Olympus IX81 confocal microscope (60x objective) and FluoView500 software.

### Blocking of CD11b/CD18 and CD11c/CD18 by antibodies

The role of CD11b/CD18 and CD11c/CD18 in the adhesion to fibrinogen was analyzed by comparing the adhesive properties of MDMs and MDDCs treated with either 50 μg/ml anti-CD11b antibody (monoclonal mIgG1 clone ICRF44, Biolegend) or 50 μg/ml anti-CD11c antibody (monoclonal mIgG1 clone 3.9, Biolegend). Both antibodies are specific for the ligand binding domain of the corresponding integrin and were used in sterile, azide-free form at saturating concentration previously titrated by flow cytometry. The effect of receptor specific antibodies was compared to samples incubated with an isotype matched control antibody (mouse IgG1 clone MOPC-21, Biolegend). To exclude Fc-receptor mediated binding of the blocking monoclonal antibodies we used an Fc-receptor blocking reagent (Miltenyi Biotech) prior to the antibody treatment. Cells were incubated with the receptor-specific antibodies for 30 minutes at 4°C then used in the functional studies without washing, since unblocked integrins are known to recycle and appear on the cell surface from an intracellular pool [35], that would decrease the efficiency of receptor blocking.

### Analysis of adhesion

#### Analysis of adhesion by confocal microscopy

The wells of a CELLview cell culture dish with glass bottom (Greiner Bio-One, Kremsmünster, Austria) were coated with 10 μg/ml fibrinogen (Merck, Darmstadt, Germany) in PBS for 1 hour at 37°C, free surfaces were blocked with synthetic copolymer poly (L-lysine)-graft-poly (ethylene glycol) (PLL-g-PEG, SuSoS AG, Dübendorf, Switzerland) for 30 minutes at 37°C.

The density of fibrinogen was titrated to a concentration that supported the highest number of adherent cells. In a preliminary experiment we found that the number of fibrinogen ligands on the adhesive surface is comparable with the total amount of receptors found on monocytes (50x10^3^ CD11b and 7x10^3^ CD11c molecules on their surface) [9,11]. Since macrophages and dendritic cell have far more of these receptors on their surface (in a range of 200-300x10^3^), we propose that this ligand density is suitable for the assessment of receptor usage.

After washing the wells twice with PBS, 4x10^4^ cells, previously incubated with the blocking antibodies as described above, were transferred to the wells and let to adhere for 30 minutes at 37°C in a CO_2_ incubator. After the incubation, samples were fixed with 2% paraformaldehyde (Sigma-Aldrich) for 10 minutes and unbound cells were removed by washing 2 times with PBS. The number of adhered cells was determined by staining the nuclei with Draq5 (BioLegend) diluted 1000x (5 μM) in PBS and incubated for 15 minutes at RT. Samples were analyzed by Olympus IX81 confocal microscope (10x objective) and FluoView500 software. 10 representative fields were scanned in each well, and the number of cells was determined using ImageJ software.

#### Measuring the force of adhesion with the computer-controlled micropipette

The force of adhesion on a fibrinogen coated surface was firstly compared between untreated control cells and cells treated with LPS for 24 hours. Then, to determine the participation of CR3 and CR4, the adhesion force was measured after blocking either CD11b or CD11c on LPS treated MDMs and MDDCs.

Single cell adhesion force was analyzed with an imaging-based automated micropipette (CellSorter, Budapest, Hungary) as described previously [36,37]. Briefly, a 5 mm by 5 mm PDMS insert (SYLGARD 184, Dow Corning, Midland, Michigan, USA) was placed in a Petri dish and the surface was coated with 10 μg/ml fibrinogen in PBS for 1 hour at 37°C. Dishes were washed twice with PBS and the surface was blocked with 100 μg/ml synthetic copolymer PLL-g-PEG (SuSoS AG), in order to inhibit non-specific cell adhesion, for 30 minutes at RT. Dishes were incubated at 4°C overnight, then washed with PBS. Afterwards, 2x10^4^ cells in RMPI-10% FCS were placed onto the coated surface and incubated for 30 minutes at 37°C in a 5% CO_2_ atmosphere. Cultures were washed 2 times with Hanks’ Balanced Salt solution with sodium bicarbonate without phenol red buffer (HBSS, Sigma-Aldrich) to remove floating cells. Region of interest (ROI) of the Petri dish was scanned by a motorized microscope (Zeiss Axio Observer A1) equipped with a digital camera (Qimaging Retiga 1300 cooled CCD). Cells were automatically recognized by the CellSorter software. To minimize the duration of the measurement, the shortest path of the micropipette was calculated by software [38]. Individual cells were visited and probed by the glass micropipette. The micropipette with an aperture of 70 μm approached the surface to a distance of 10 μm. Vacuum was generated in a standard syringe connected to the micropipette via a high speed normally closed fluid valve. To probe cell adhesion the valve was opened for 20 ms generating a precisely controlled fluid flow and corresponding hydrodynamic lifting force acting only on the targeted cell. The hydrodynamic lifting force was calculated by running computer simulation solving the Navier-Stokes equation in a geometry corresponding to the experimental setup [37]. After each cycle of the adhesion force measurement the ROI of the Petri dish was scanned again, and the vacuum was increased to the next level. The micropipette visited again each location determined after the initial scan. Suction force was increased as long as most of the cells were removed. The number of cells were counted in the images before and after each cycle of the adhesion force measurement and calculated the ratio of still adhering cells of the population placed onto the surface at the beginning of the experiment.

### Analysis of the contact surface by confocal microscopy

For the contact surface analysis 10^4^ cells were plated on a fibrinogen coated surface as described above.

#### Staining of the actin cytoskeleton and podosome counting

The actin cytoskeleton of adhered cells was stained with phalloidin-Alexa488 (Molecular Probes, Invitrogen, Eugene, Oregon, USA) diluted 1:80 in PBS-0.1% Triton X-100 (Reanal, Budapest, Hungary) for 15 minutes, then washed 2 times with PBS. Nuclei were stained with Draq5 (BioLegend) diluted 1:1000 (5 μM) in PBS for 15 minutes. Samples were analyzed by an Olympus IX81 confocal microscope (60x objective) applying Fluowiev500 confocal workstation.

The number of podosomes was counted using the method described by Cervero et al. [39]. Briefly, the podosome number and contact zone area of the cells was determined using ImageJ software. Podosome density of 40–60 cells/sample was calculated as the number of podosomes per 100 μm^2^ cell covered area.

#### Staining of CD11b/CD11c on adherent cells

Fixed samples were permeabilized with 0.1% Tween-20 (Reanal) in PBS for 20 minutes at 37°C. The permeabilizing solution was replaced with a blocking solution: 0.2% gelatin (Merck) and 0.1% Tween-20 in PBS and cells were incubated for 1 hour at 37°C. Cells were incubated with the primary antibodies overnight at 37°C, followed by the secondary antibodies for 2 hours at 37°C. The antibodies were diluted in the blocking solution. Cells were washed with 0.1% Tween-20 in PBS 3 times for 5 minutes each before adding the secondary antibody. Antibodies used for staining: anti-CD11b (EP1345Y, Abcam, Cambridge, UK), anti-CD11c (EP1347Y, Abcam), goat anti-rabbit IgG-Alexa647 (Molecular Probes). After the secondary antibody staining cells were washed 3 times for 5 minutes each with 0.1% Tween-20 in PBS. The actin cytoskeleton was stained with phalloidin-Alexa488 (Molecular Probes) 1:80 diluted in 0.1% Tween-20 PBS for 45 minutes at 37°C. Samples were analyzed by an Olympus IX81 confocal microscope (60x objective). For better visibility the brightness and contrast of images was adjusted using Adobe Photoshop CS3.

### Analysis of transmigration

Migration of both untreated and LPS treated (24 hours) cells was analyzed using 24 well transwell plates (polycarbonate membrane with 5 μm pore, Corning, Corning, NY, USA). 10^5^ cells in 100 μl RPMI-1640 medium were plated in the upper chamber, and the lower chamber was filled with 600 μl of chemoattractant solution or medium (negative control). For the untreated cells 62.5 nM N-Formylmethionine-leucyl-phenylalanine (FMLP, Sigma-Aldrich) was used as a chemoattractant and the migration was stopped after 1 hour. For the LPS treated cells we used 200 ng/ml CCL19 (BioLegend), and the migration was stopped after 2 hours for MDDCs and 4 hours for MDMs. At the end of the incubation time, EDTA was pipetted to the lower chamber without removing the membrane, at a final concentration of 12.5 mM. After 5 minutes at 37°C cells were resuspended from the lower chamber and the bottom of the membrane, placed on ice and counted immediately. The number of transmigrated cells was determined in a volume of 250 μl/sample by flow cytometry (Cytoflex, BC).

### Statistics

Statistical tests (specified at the data presentation) were performed with GraphPad Prism 5 software, p<0.05 was considered significant. One-way ANOVA was used to compare three or more sets of measurements when only one factor was changed during the experiment. Two-way ANOVA was employed when the effect of two factors were examined simultaneously.

## Results

### Under inflammatory conditions CR3 and CR4 is expressed differently on MDMs and MDDCs

**Fig 1:**
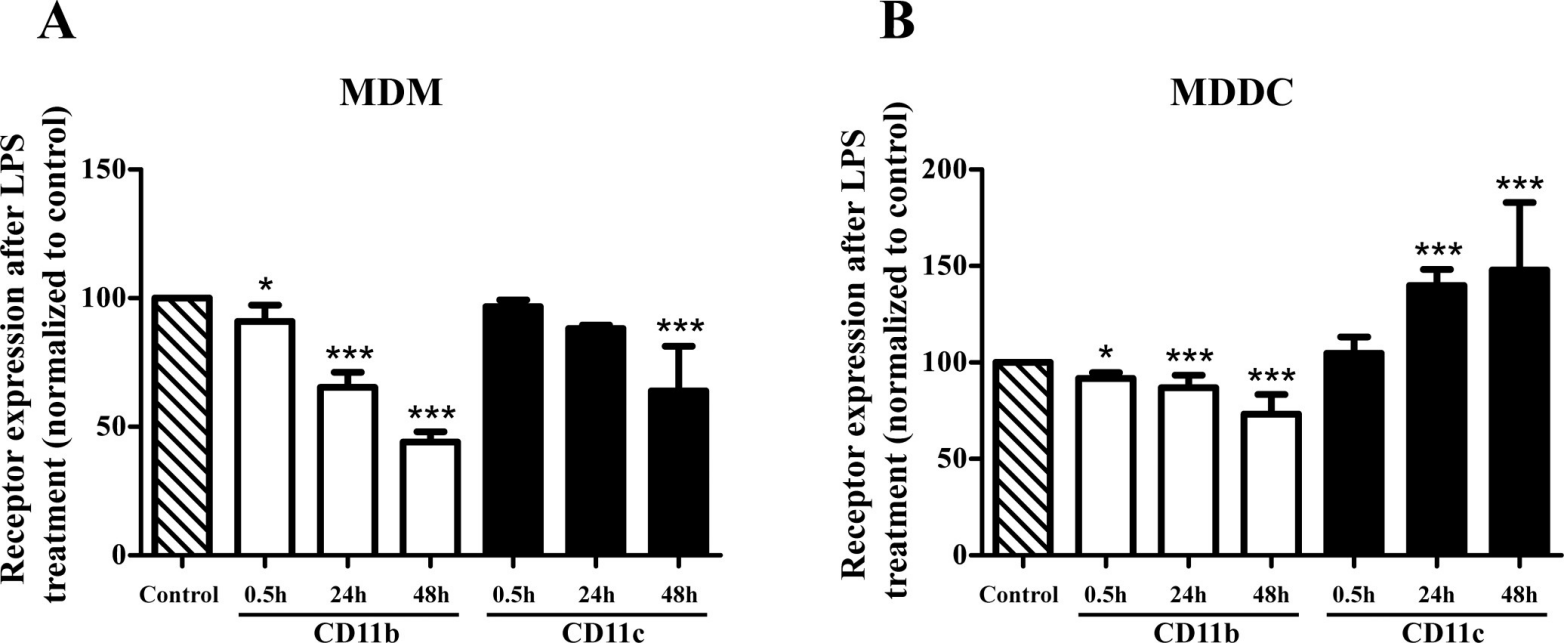
Changes in the expression of CR3 and CR4 after LPS induced activation. The surface expression of CR3 and CR4 was measured at different time points by flow cytometry. The effect of 100 ng/ml LPS treatment was compared to the appropriate untreated control sample at each time point, shown as 100%. For MDMs (A) results of 3 donors (mean ± SD), for MDDCs (B) 4 donors’ results (mean ± SD) are shown. Two-way ANOVA with Bonferroni post-test was used to determine significant differences compared to control, * = p < 0.05; ** = p < 0.01; *** = p < 0.001.

The expression of CR3 and CR4 was monitored by flow cytometry at different time points during the LPS induced cell activation. Comparing the amount of CR3 and CR4 on the cell surface we found that LPS treatment alters their expression differently on MDMs and MDDCs (Fig 1). Whereas on MDMs the expression of both CR3 and CR4 decreases, on MDDCs only the expression of CR3 is diminished, CR4 appears at a significantly elevated level. Cytometry profiles of CR3 and CR4 expression after LPS treatment are shown in S1 Fig.

### LPS treatment enhances β_2_-integrin cycling in both MDMs and MDDCs

**Fig 2:**
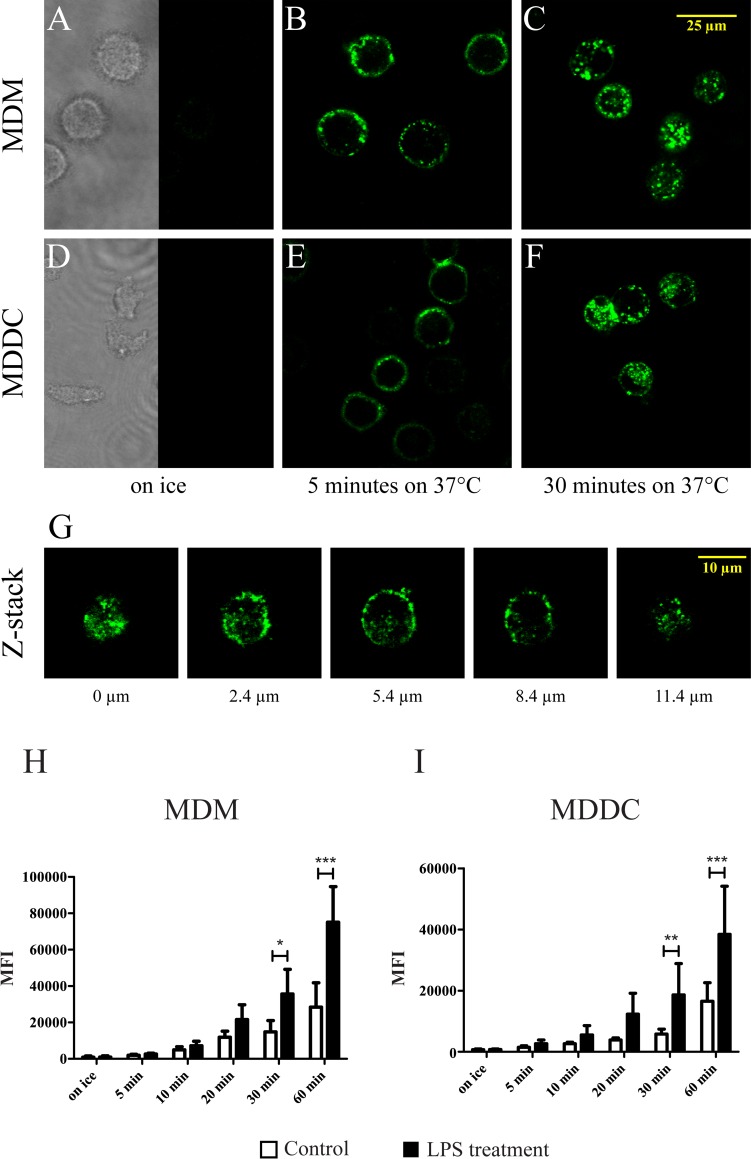
Localization and conformational state of β_2_-integrins. The conformational state of β_2_-integrins was analyzed by confocal microscopy (A-G) and flow cytometry (H-I). Cells were incubated with the Alexa488 labelled mAb24 antibody for 20 minutes on ice, then, without washing, cells were moved to a 37°C incubator for the indicated period of times, with or without 100 ng/ml LPS. The staining of unstimulated MDMs (A-C) and MDDCs (D-F) shows an even distribution of active β_2_-integrins in the cell membrane after 5 minutes of incubation at 37°C, whereas after 30 minutes the labelled receptors appeared in the cytoplasm of the cell. Representative z-stack slices of MDDC (G) confirm the internalization of β_2_-integrins after 30 minutes at 37°C (the bottom of the cell is shown as 0 μm). Representative pictures are shown out of 3 donors’ results. The effect of LPS treatment on the activation state of β_2_-integrins was analyzed by flow cytometry. Mean fluorescence intensity (MFI) data of 4 donors are presented for MDMs (H) (mean ± SD) and 5 donors for MDDCs (I) (mean ± SD). Two-way ANOVA with Bonferroni post-test was used to determine significant differences, * = p < 0.05; ** = p < 0.01; *** = p < 0.001.

The ligand binding affinity of β_2_-integrins is regulated by activation dependent conformational changes, when these receptors switch between an inactive bent and an activated extended structure. The conformational state of the receptors can be checked using the mouse monoclonal mAb24 antibody, which is specific for an activation epitope in the I-like domain of the β_2_ chain (CD18). This determinant is revealed only in the activated, high affinity receptor with an open conformation; therefore, this antibody does not bind to the receptors of cells kept on ice (Fig 2A, 2D, 2H and 2I). Cells were incubated with the Alexa488 labelled mAb24 antibody for 20 minutes on ice, then, without washing, cells were moved to a 37°C incubator. Under the experimental conditions that were used for our functional studies, the receptors were in an active, open conformation, confirmed by the binding of the mAb24 antibody.

Confocal microscopy images showed an even distribution of β_2_-integrins in the cell membrane after 5 minutes of incubation at 37°C, whereas after 30 minutes the labelled receptors started to accumulate inside the cell (Fig 2A–2G). The internalization of β_2_-integrins was confirmed with z-stack analysis (Fig 2G). Integrins are known to have a fast turnover between the cell surface and intracellular pools, leading to the appearance of unlabeled receptors on the cell surface, that bind the mAb24 antibody, increasing the fluorescence intensity with time. When 100 ng/ml LPS was added to the cells during the 37°C incubation time, the mean fluorescence intensity (MFI) was significantly higher, proving that LPS enhances the activation and turnover of β_2_-integrins (Fig 2H and 2I).

### Under inflammatory conditions both CR3 and CR4 participate in the adhesion to fibrinogen

**Fig 3:**
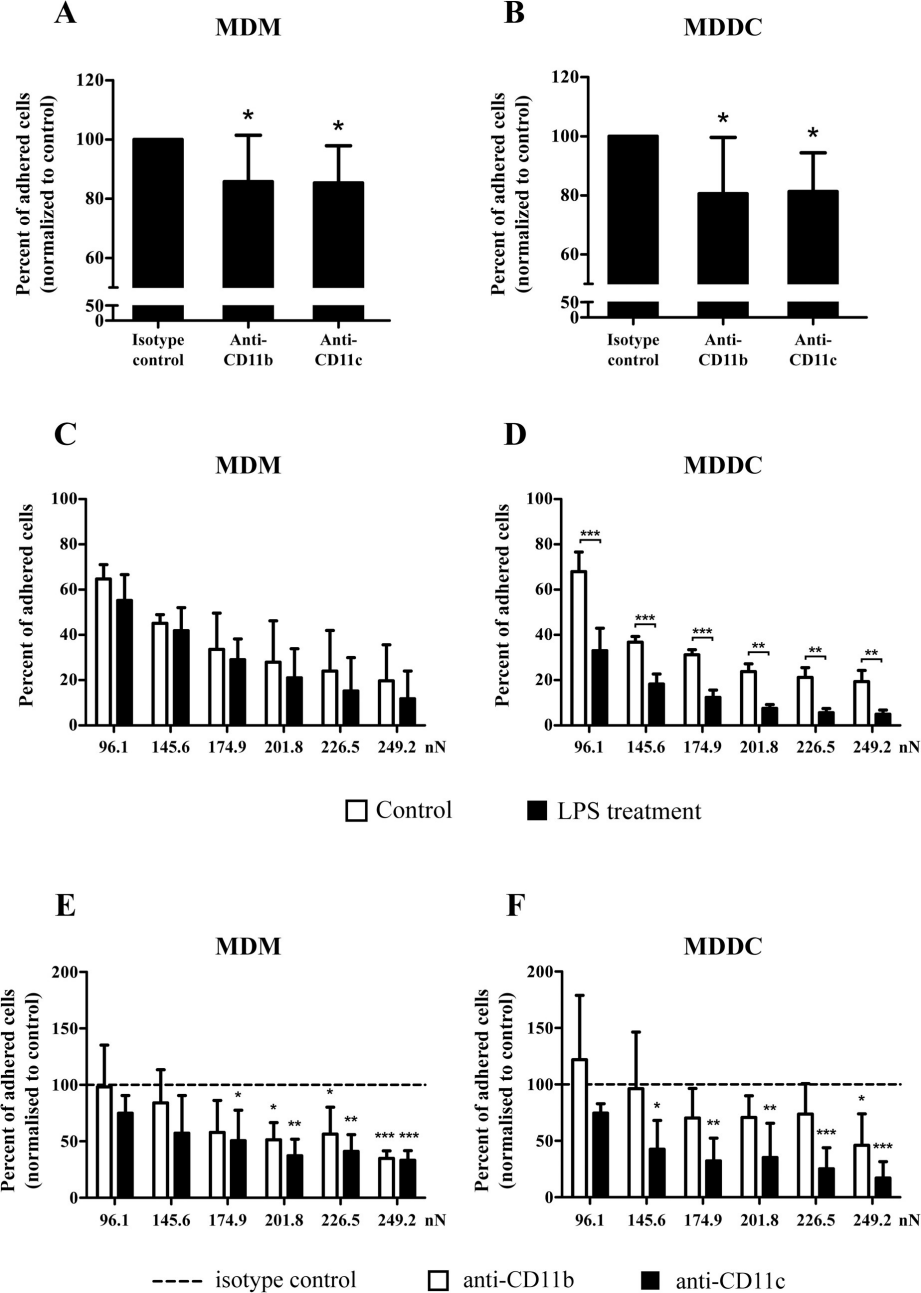
The effect of the β_2_-integrins’ ligand binding site specific antibodies and LPS on the adhesion to fibrinogen. (A-B) The adhesion capacity of LPS activated MDMs (A) and MDDCs (B) was measured with the classical adhesion assay. The cells were treated with ligand binding site specific blocking antibodies (anti-CD11b or anti-CD11c), then let to adhere on a fibrinogen coated surface. The number of adhered cells was normalized to the isotype control antibody treated samples, shown as 100%. Results of 7 donors are shown as mean ± SD. One-way ANOVA with Tukey’s post-test was used to determine significant differences compared to control, * = p < 0.05. (C-F) The force of cell attachment was measured with a computer-controlled micropipette using vacuum induced fluid flow. The percent of adhered cells was calculated by dividing the number of cells remaining on the surface after applying a given vacuum, by the number of cells originally contained in the population. The impact of LPS treatment on the force of adhesion is shown on panel C-D. The effect of blocking antibody treatment (anti-CD11b: white columns; anti-CD11c: black columns) on the adhesion of LPS treated cells is shown on panel E-F. The percent of adhered cell was normalized to the isotype control antibody treated samples (shown as 100%, represented by the dashed line). For MDMs (C, E) results of 3 donors (mean ± SD), for MDDCs (D, F) 4 donors’ results (mean ± SD) are shown. Two-way ANOVA with Bonferroni post-test was used to determine significant differences compared to control. * = p < 0.05; ** = p < 0.01; *** = p < 0.001.

We set out to study the participation of CR3 and CR4 in the adhesion of MDMs and MDDCs to a fibrinogen coated surface first by a classical method, where the number of adherent cells on a fibrinogen coated surface was counted. To distinguish the function of CR3 and CR4 in adhesion under inflammatory conditions, cells were treated with either anti-CD11b or anti-CD11c antibody prior to the assay. Unbound cells were washed away before counting. We found that in the case of LPS treated MDMs and MDDCs both CR3 and CR4 participated in cell adhesion, as blocking antibodies equally decreased the number of adherent cells compared to the isotype control antibody treated samples (Fig 3A and 3B). The low inhibition observed with this method can be attributed to the fibrinogen binding ability of other receptors, namely, α_V_β_3_ and α_5_β_1_ present on myeloid cells [40–42] and the high recyclization rate of β_2_ integrins (shown on Fig 2) that might lower the efficiency of antibody blocking. However, the inhibition by the specific antibodies proves that CR3 and CR4 contribute to the adhesion to fibrinogen equally.

### LPS induced maturation decreases the force of adhesion significantly in MDDCs

In this set of experiments, we evaluated cell adhesion to fibrinogen using a state-of-the-art biophysical method, which allows the measurement of the force of cell attachment with a computer-controlled micropipette. Cells were let to adhere on a fibrinogen coated surface, and their adhesion force was assessed by trying to pick them up with the micropipette using vacuum induced fluid flow. The pick-up process was repeated several times with increased vacuum, and cells remaining on the surface were counted after each cycle. Applied vacuum was converted to force (nN) based on computer simulations. Employing the micropipette, we observed a significantly reduced adhesion force for LPS treated MDDCs (Fig 3D). In the case of LPS activated MDMs we observed only a slight decrease in the strength of attachment compared to the untreated cells (Fig 3C).

### CR4 is prominently involved in the strong attachment of MDMs and MDDCs to fibrinogen

To differentiate the role of CR3 and CR4 in the adhesion to fibrinogen we blocked either CD11b or CD11c by ligand binding site specific monoclonal antibodies. Experimental data are presented as the ratio of blocking antibody treated adherent cells compared to the isotype control treated cells. Previously we proved, that under physiological conditions only the blocking of CD11c decreased the adhesion force of MDMs and MDDCs, CD11b did not play a role in cell attachment [11]. Here we demonstrate, that during inflammatory conditions, both receptors become involved in adherence, however the blocking of CD11c resulted in a weaker attachment of MDDCs compared to the anti-CD11b treated samples (Fig 3E and 3F).

### MDDCs lose their podosomes during LPS induced maturation

**Fig 4:**
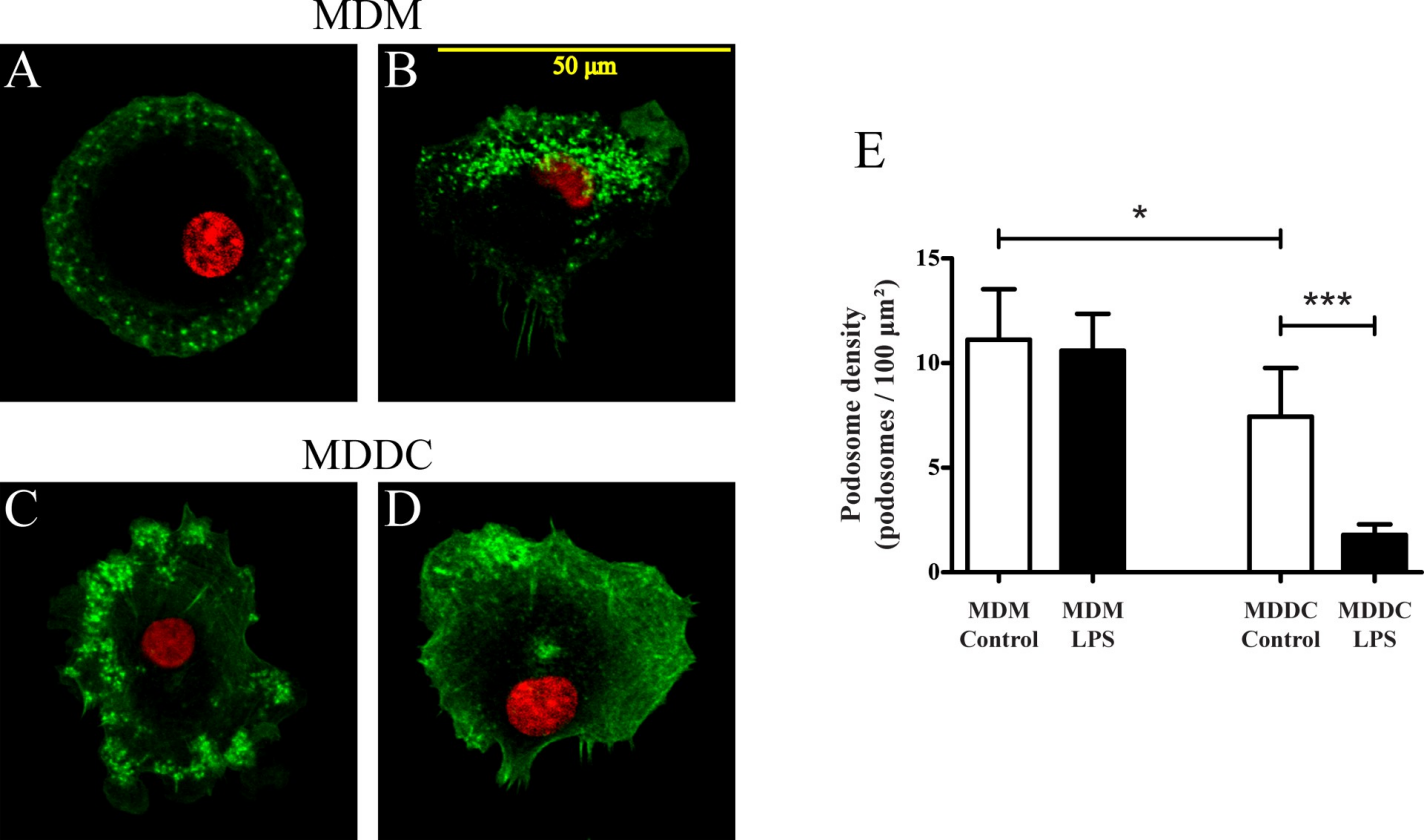
Changes in the arrangement of podosomes in the contact surface of adherent cells upon LPS treatment. The actin cytoskeleton of adherent cells was stained with phalloidin-Alexa488 (shown in green), nuclei were stained with Draq5 DNA dye (shown in red). Both untreated (A) and LPS treated (B) MDMs form podosomes during adhesion, whereas only untreated, immature MDDCs (C) contain these structures, LPS treated mature MDDCs (D) lose their ability for podosome formation. The number of podosomes and cell area were measured on 40–60 cells/sample with ImageJ software (E), data are shown as podosome density (number of podosomes per 100μm^2^ of cell covered area). One-way ANOVA with Tukey’s post-test was used to determine significant differences compared to control, * = p < 0.05, *** = p < 0.001. Representative microscopy pictures (A-D) are shown out of 5 (MDMs) or 7 (MDDCs) donors’ results and podosome density data (E) are shown as mean ± SD of the above data.

Podosomes are adhesive structures known to mediate short-lived adhesion spots that are formed and quickly remodeled during migration. Considering their importance in cell attachment and movement, we set out to study their formation after LPS treatment by staining the actin cytoskeleton with phalloidin-Alexa488. Cells were let to adhere on a fibrinogen coated surface and nuclei were stained with Draq5 DNA dye. As it is demonstrated in Fig 4, panel C and D, LPS treatment induced the loss of podosomes in dendritic cells, whereas activated macrophages preserve these structures (Fig 4A and 4B). To quantify these differences, the number of podosomes was counted based on the phalloidin staining of podosome actin cores using ImageJ software (Fig 4E). These data confirmed that the number of podosomes is unchanged in MDMs, but drastically decreased in MDDCs after LPS treatment. This remarkable difference in the ability of podosome formation explains the greater reduction in adhesion force of MDDCs compared to MDMs after LPS treatment.

Previously we demonstrated that podosomes can be arranged in various patterns in different cell types. In the case of MDMs we observed a belt-like or even distribution, whereas in MDDCs clusters of podosomes appeared [12]. Here we show, that in addition to a difference in podosome arrangement, the number of these structures is significantly lower for MDDCs than MDMs (Fig 4E).

### Differential distribution of CD11b and CD11c on the contact surface of adherent cells after LPS treatment

**Fig 5:**
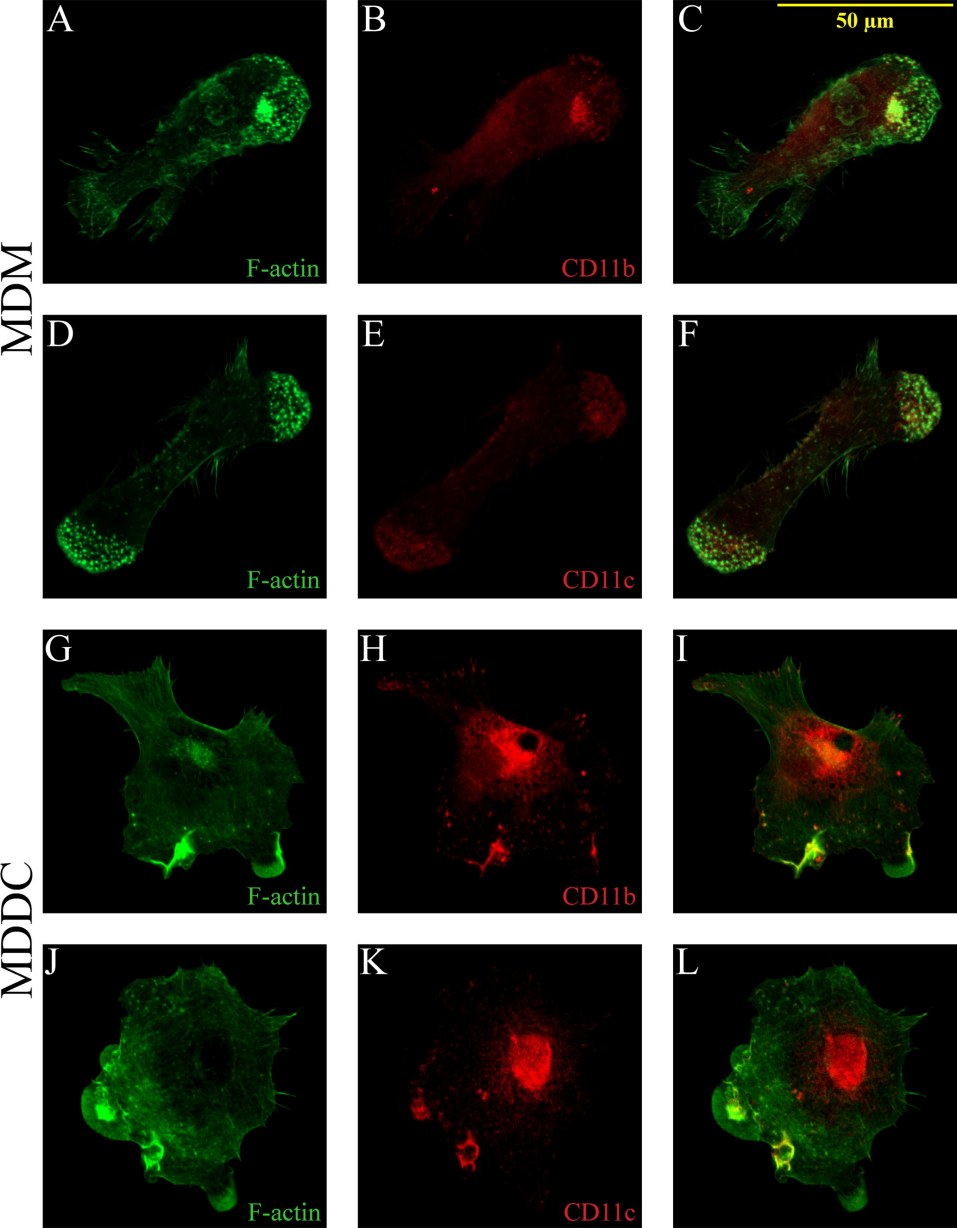
Localization of CD11b and CD11c on the contact surface changes upon LPS-treatment. MDMs (A–F) and MDDCs (G–L) were plated to a fibrinogen coated surface, followed by staining with phalloidin-Alexa488 (A,D,G,J), anti-CD11b (B,H) or anti-CD11c (E,K). Merged images are shown in C,F,I,L. CD11b and CD11c are located around the actin core of podosomes in LPS treated MDMs. In the case of LPS treated mature MDDCs, with the loss of podosomes, CD11b and CD11c is concentrated in the cell body around the nucleus, and in membrane ruffles on the leading edge. Images of one representative experiment out of 3 are shown.

Previously we demonstrated the presence of both CD11b and CD11c in the podosome adhesion ring on the contact surface of MDMs and MDDCs during physiological conditions [12]. Under inflammatory conditions or upon maturation, parallel with the changes in the functions of various cells, podosomes might be lost. In our present study we found that LPS activated MDMs retain their ability to form podosomes with CD11b and CD11c around the actin core (Fig 5A–5F). In LPS treated mature MDDCs however, with the loss of podosomes, CD11b and CD11c is concentrated in the cell body around the nucleus, and in membrane ruffles on the leading edge (Fig 5G–5L).

### Both CR3 and CR4 participate in the migration of MDMs and MDDCs

The migration capacity of MDMs and MDDCs was tested using a transwell assay with a 5 μm pore size polycarbonate membrane. To study the involvement of CD11b and CD11c, ligand binding site specific antibodies were employed, and the number of transmigrated cells were counted by flow cytometry.

**Fig 6:**
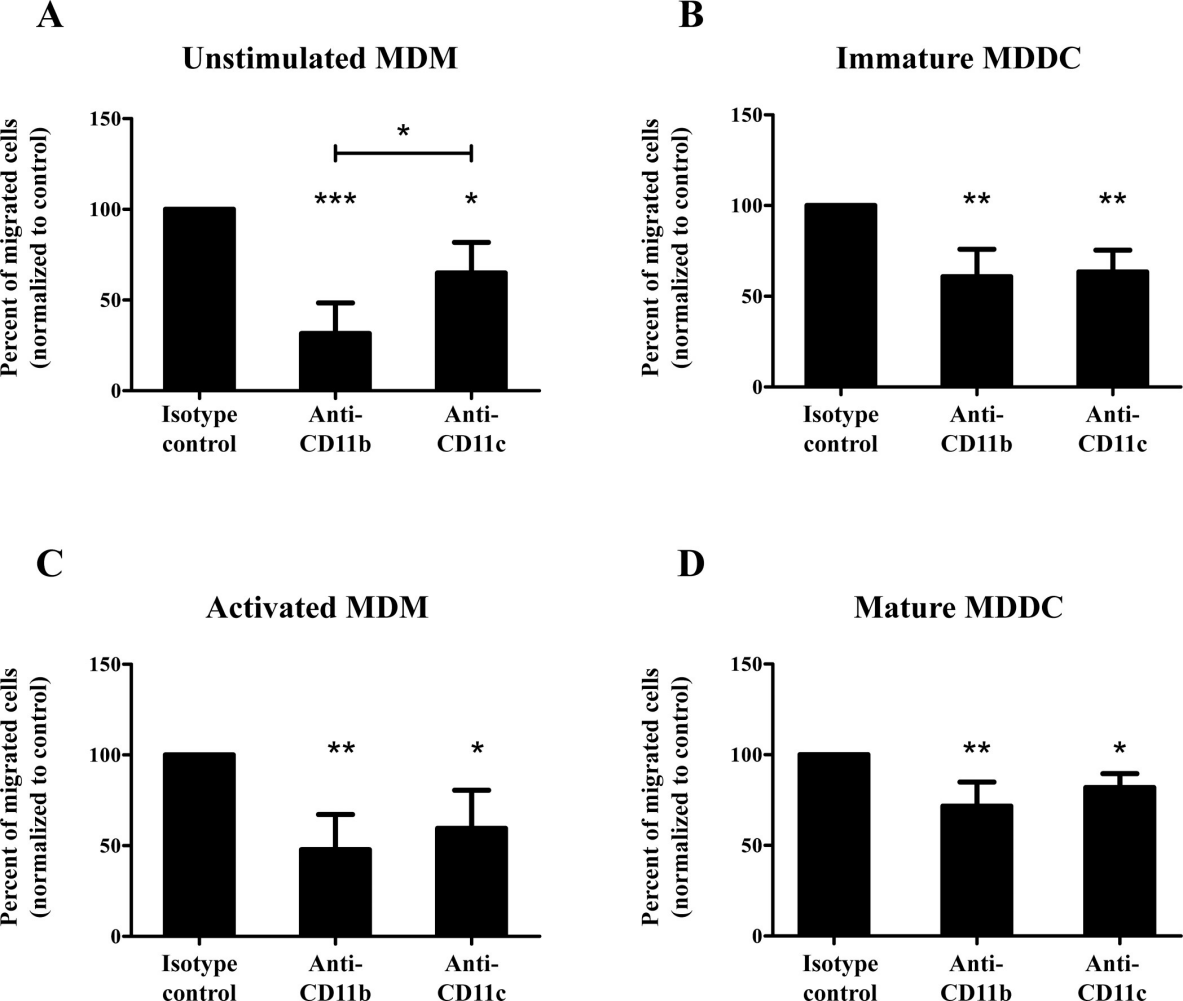
Analysis of the migration of MDMs and MDDCs using FMLP or CCL19 as a chemoattractant. FMLP was used as a chemoattractant for unstimulated MDMs (A) and immature MDDCs (B), and CCL19 was applied for activated MDMs (C) and mature MDDCs (D). Cells were treated with either anti-CD11b or anti-CD11c ligand binding site specific antibodies before migration, and the number of transmigrated cells were counted by flow cytometry after detachment using EDTA. The number of migrated cells was normalized to the isotype control antibody treated samples, shown as 100%. One-way ANOVA with Tukey’s post-test was used to determine significant differences compared to control, * = p < 0.05; ** = p < 0.01, *** = p < 0.001.

Unstimulated MDMs and immature MDDCs are known to migrate towards N-formylated peptides as they express formyl peptide receptors (FPRs). As a synthetic ligand N-Formylmethionyl-leucyl-phenylalanine (FMLP) was used, which acts similarly on FPRs as the natural ligands. As shown in Fig 6A and 6B, both anti-CD11b and anti-CD11c reduced the number of cells able to migrate through the membrane. For MDMs the blocking of CR3 resulted in a significantly stronger inhibition than the blocking of CR4, while in the case of MDDCs the extent of inhibition was the same for both receptors.

The expression of chemokine receptors and adhesion molecules, and consequently migration capacity, is influenced by the activation status of immune cells. The CCR7 chemokine receptor appears on macrophages and DCs upon activation, therefore we used its ligand, CCL19 as a chemoattractant to study migration under LPS induced inflammatory conditions. Results obtained from the transwell assay show that both CR3 and CR4 participate in the migration of the LPS treated cells (Fig 6C and 6D), and similarly to the untreated cells, blocking with anti-CD11b results in a somewhat stronger inhibition, when compared to the effect of anti-CD11c, particularly in the case of unstimulated MDMs.

## Discussion

Macrophages and dendritic cells are essential in the recognition of invading pathogens and the initiation of immune responses. Both cell types are professional antigen presenting cells, but they have different roles in several aspects. Dendritic cells migrate to lymph nodes after antigen uptake to initiate an adaptive immune response by presenting these antigens to naïve T-cells [43]. The main function of macrophages is to migrate within the peripheral tissues to the site of infection, where they take up the antigen and eliminate pathogens by phagocytosis, production of reactive intermediates and the establishment of an inflammatory milieu by cytokine secretion [44].

After the lysis of Gram-negative bacteria, glycolipids from the outer membrane are released, that serve as potent proinflammatory stimuli for myeloid cells. In vivo studies show that the administration of bacterial lipopolysaccharides (LPS) induces acute inflammation through eliciting the migration, cytokine production and ROS release of neutrophils and macrophages in mice [45,46], and it also initiates the maturation of dendritic cells [47]. **S**tudies on the environmental effects on respiratory functions of asthma patients show that the inhalation of air-borne endotoxins, for example in grain or house dusts, increases the severity of respiratory diseases [48–50]. Endotoxins in the lungs induce an inflammatory reaction, that is characterized by the influx of neutrophils and an elevated fibronectin production [51]. Additionally, LPS is a major inducer of tissue injury and sepsis after a Gram-negative bacterial infection [52]. The overproduction of LPS-induced inflammatory mediators by mieloid cells is the leading cause of mortality during septic shock [53,54].

LPS is known to bind to Toll-like receptors and β_2_-integrins that promote receptor activation and cell adhesion. Wright et al. identified an LPS binding site in the CR3 α chain that was distinct from the location of iC3b and fibrinogen binding [55]. Wong et al. identified two putative LPS binding sites in the β_2_ I-like domain [56] that would explain why LPS binding to either LFA-1, CR3 or CR4 induces an inflammatory response [57–59]. CR3 was also shown to become associated with CD14 after LPS treatment on neutrophils [60], and Ingalls et al. suggested that it might use the same adaptors for LPS signaling as CD14 [61].

Antigen uptake and proinflammatory stimuli induce macrophage activation and dendritic cell maturation involving changes in the expression of costimulatory molecules, chemokine and phagocytic receptors [62]. Regarding neutrophil granulocytes, it is known that upon stimulation by LPS they quickly, but transiently upregulate the expression of CR3 from intracellular granules, and the receptors on the cell surface switch to high affinity conformation, both events contributing to enhanced adhesiveness [63,64].

In the present work we monitored the changes in the expression of CD11b and CD11c on myeloid cells upon LPS stimulation and found that the appearance of these receptors is time dependent. Namely, on MDMs and MDDCs treated with LPS for 30 minutes the amount of CD11b slightly decreased, while the expression of CD11c did not change. After longer periods however,–i.e. 24 and 48 hours later—the amount of CD11b decreased on both cell types. At the same time, the quantity of CD11c increased in MDDCs but was lower in MDMs, suggesting a cell type specific regulation of β_2_-integrin expression. Investigating their conformation, we found a significantly elevated number of active cell membrane receptors after 30 minutes of LPS treatment–confirmed by binding mAb24 -, due to the increased turnover of the receptors. Since this antibody is specific for an activation epitope in all β_2_-integrins, it can recognize LFA-1 (CD11a/CD18 or α_L_β_2_) too. The endocytosis rate of this receptor however has been shown to be very low compared to CR3, furthermore it is not influenced by the stimulating agents [35]. Consequently, the increase in internalization and recycling speed observed by us can be attributed to CR3 and CR4.

These results demonstrate that the amount of cell surface CR3 and CR4 does not increase immediately after LPS treatment, however their activation status and recycling speed changes. Integrin activation and recycling are most likely linked processes. Arjonen et al. showed that active β_1_-integrins have a higher endocytosis rate, whereas receptors in an inactive state are rapidly recycled to the plasma membrane [65]. Based on our data we propose that under inflammatory conditions β_2_-integrin activation triggers the endocytosis as well as faster recycling of these receptors. Thus, the higher endocytosis rate of CR3 in the case of LPS activated cells explains the decrease in cell surface expression after 30 minutes.

Integrins are known to mediate cell adhesion, spreading and migration through the establishment of cell-cell and cell-extracellular matrix connections [66,67]. Our group focuses on dissecting the individual roles of the β_2_-integrins CR3 and CR4, querying the assumption that they have overlapping, or even identical functions. Early studies on human granulocytes and monocytes proved that LFA-1, CR3 and CR4 all contribute to endothelial adhesion [68,69], and that the cell type and the stimuli used can influence the participation of these receptors. Georgakopoulos et al. also showed, that the involvement of CR3 and CR4 in adhesion to fibrinogen is dependent on the experimental conditions, namely the type of stimuli and culture conditions of human monocytes [70]. Earlier we demonstrated that in the absence of inflammatory stimulus CR4 plays a dominant role in the adhesion to fibrinogen [11].

Here, we studied the adhesive capacity of MDMs and MDDCs after LPS stimulation. We used both the classical, end-point adhesion assay and a computer-controlled micropipette method capable of measuring the force of adhesion [71]. When applying ligand binding site specific antibodies, we found that both anti-CD11b and anti-CD11c treatment weakened the adhesion to fibrinogen coated surfaces, as verified by the decreased number of adherent cells as well as the force of attachment. Importantly however, while both receptors participated in adhesion, the force of attachment was significantly lower for the anti-CD11c treated cells in the case of MDDCs, proving a dominant role for CR4 on this cell type.

This differential participation of CR3 and CR4 in adhesion confirms our previous hypothesis [11], namely that ligand availability and the number of cell surface receptors regulates receptor utilization. Based on the absolute number of cell surface receptors [9,11] we proved that there is a competition for accessible fibrinogen ligands between CR3 and CR4 on MDMs and MDDCs, where both receptors are present in high amounts. On monocytes however, both receptors took part in adherence, because their amount is comparable with the number of fibrinogen ligands. Since LPS treatment altered the cell surface expression of these receptors differently on MDMs and MDDCs, we detected a change in receptor usage. On MDMs the number of both receptors decreased upon LPS treatment, allowing the ligand binding by both CR3 and CR4. In the case of MDDCs the ratio of cell membrane receptors shifts in favor of CR4, strengthening its dominant role.

Emerging data on podosomes highlight their importance in myeloid cell adhesion and migration. Considering the differences in immune functions of macrophages and dendritic cells we compared the formation and β_2_-integrin composition of podosomes after inflammatory stimuli on these cell types. During LPS induced maturation dendritic cells go through cytoskeletal changes, losing their capacity for phagocytosis and podosome formation [25,72,73]. Van Helden et al. showed that in human DCs the loss of podosomes is rapidly (5–10 minutes) induced by prostaglandins, but it takes at least 16 hours after LPS treatment through the engagement of TLR4 and the induction of prostaglandin production. At the same time the migration speed of mature human DCs increases without the constraint of strong podosome contacts [34,74]. In mouse DCs however, LPS induces a quick, but transient change. TLR signals enhance phagocytosis and antigen sampling, during which cells switch from podosome formation to focal contacts, losing their ability to migrate. Two hours after LPS stimulus podosomes become prominent again, and the migration speed of DCs is restored [33,75]. In this study we confirm, that after 24 hours of LPS treatment MDDCs do not form podosomes on a fibrinogen coated surface, significantly lowering the force of adherence compared to immature cells. In contrast, MDMs preserve these structures after LPS activation and there is only a slight decrease in the adhesion force measured with the computer-controlled micropipette, proving that podosomes ensure a strong attachment.

It is well established that integrin activation is necessary for cell attachment, but their coordinated inactivation is also essential to maintain directional migration [76]. Semmerich et al. showed with a constitutively active LFA-1 mutation in mice, that integrin deactivation is needed for the detachment from the adhesive surface to sustain cell movement [77]. Van Helden et al. also showed, that through the inactivation of α_5_β_1_ integrin DCs have an increased migration speed, establishing only short lived, weaker adhesion spots with the fibronectin substratum [34]. A crucial and specific role for β_2_-integrins in podosome functions has been demonstrated by others [25,26], and we have also proven, that under physiological conditions both CR3 and CR4 are located in the adhesion ring of podosomes in human MDMs and MDDCs attached to fibrinogen [12]. Here we show that in the case of LPS activated MDMs, the cells become more elongated, but CD11b and CD11c are localized around the actin core of podosomes, similarly to untreated cells. On LPS matured MDDCs, with the loss of podosomes, CD11b and CD11c are concentrated in the cell body and in membrane protrusions. Although our results indicate that LPS treatment enhances β_2_-integrin activation at the early timepoints of cell activation (30–60 minutes), the loss of podosomes is associated with changes in the distribution of CR3 and CR4 that might influence the adhesive and migratory capacity of DCs.

Podosomes are proposed to participate in cell migration, but their exact role is not yet established. The experiments presented by van Helden et al. showed a faster migration speed of human DCs after podosome loss, but it was studied with an assay using a 2D surface [34]. In mice, West et al. showed with a transwell assay, that DCs do not migrate without podosomes [33]. Later van den Dries et al. provided evidence, that the dissolution of podosomes is dependent on the structure of the environment, the disassembly of podosomes is quickly accomplished on a 2D surface after prostaglandin E2 treatment, but it is inhibited in a 3D environment [78]. Considering these data and the matrix degrading ability of podosomes, these structures are more likely to be important for 3D migration and extravasation, which is slowed in their absence.

In our experiments, the migratory capacity of unstimulated and LPS treated MDMs and MDDCs was also assessed by a transwell assay using the well described chemoattractants FMLP and CCL19. We used ligand binding site specific blocking antibodies to determine the participation of CR3 and CR4 in transmigration. Although both anti-CD11b and anti-CD11c inhibited the migration of unstimulated MDMs towards FMLP, the number of migrated cells was significantly lower when CR3 was blocked. In the case of immature MDDCs both receptors took part equally. On the LPS treated MDMs and MDDCs, both receptors were found to contribute to the migration in the direction of CCL19.

Our previous studies proved a “division of labor” between CR3 and CR4 under physiological conditions [11,12]. We demonstrated that CR3 participates in the phagocytosis of iC3b opsonized *Staphylococcus aureus*, while CR4 dominates cell adhesion to fibrinogen. During inflammatory conditions the distinct role of these β_2_-integrins has not been systematically investigated before. Our present data show that after LPS treatment the expression and role of these receptors considerably changes, proving our previous hypothesis, that the number and ratio of cell surface receptors influences the outcome of cell-matrix interactions. Our findings underline the functional differences between macrophages and dendritic cells, and emphasize the essential role of podosomes for firm cell attachment.

## Supporting information

S1 Fig Changes in the expression of CR3 and CR4 after LPS induced activation. The surface expression of CR3 and CR4 was measured at different time points by flow cytometry. Representative histograms were chosen from 3 (MDMs) and 4 (MDDCs) donors’ results.
(TIF)
