# Supplementary figures and images for: The differential role of CR3 (CD11b/CD18) and CR4 (CD11c/CD18) in the adherence, migration and podosome formation of human macrophages and dendritic cells under inflammatory conditions
Source: PLoS One. 2020 May 4;15(5):e0232432. doi: 10.1371/journal.pone.0232432 (PMC7197861; doi:10.1371/journal.pone.0232432)

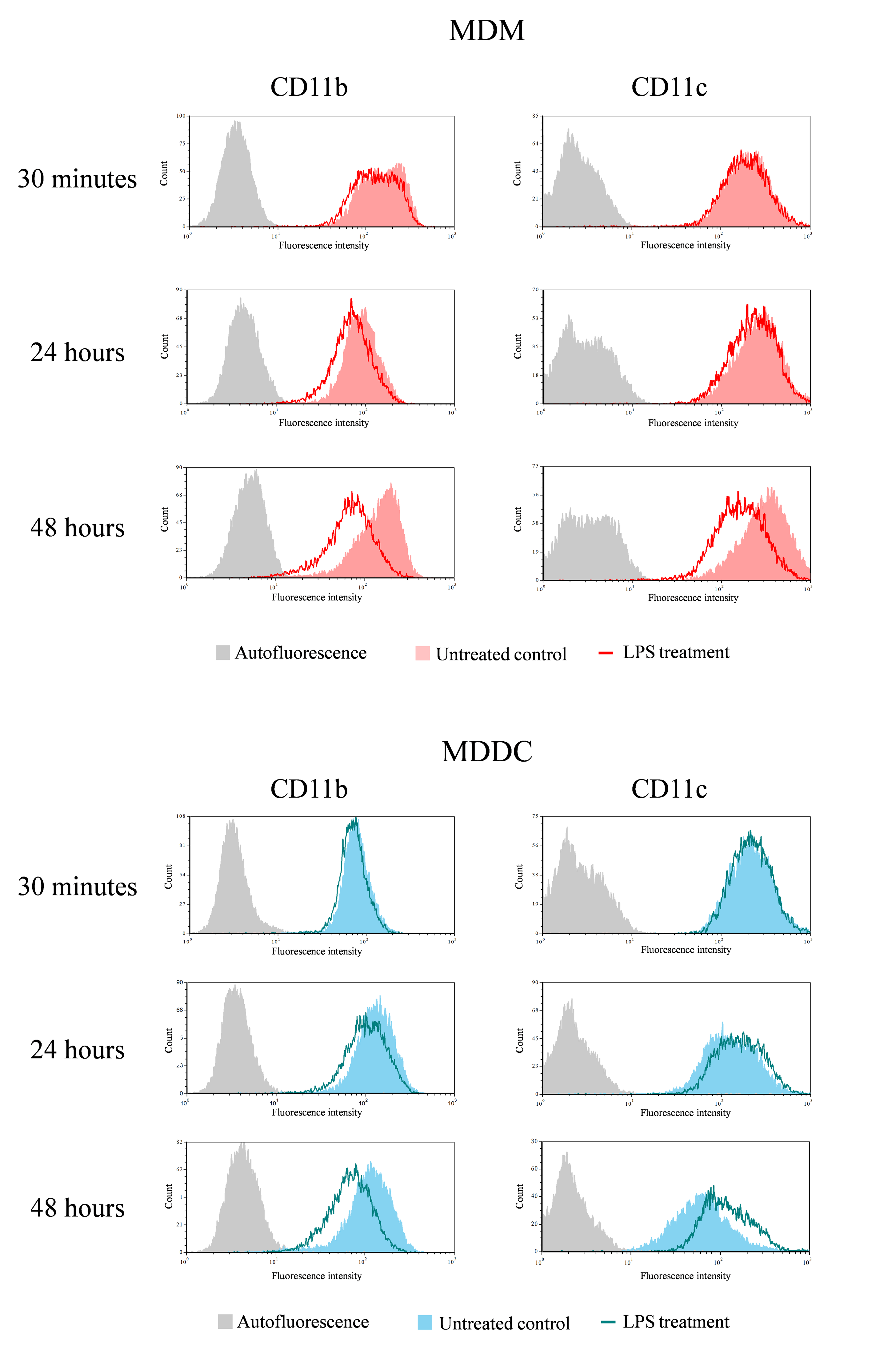

Supplement: S1 Fig — The surface expression of CR3 and CR4 was measured at different time points by flow cytometry. Representative histograms were chosen from 3 (MDMs) and 4 (MDDCs) donors’ results. (TIF) [file pone.0232432.s001.tif]
